# Sensor-based behavioral patterns can identify heat-sensitive lactating dairy cows

**DOI:** 10.1007/s00484-023-02561-w

**Published:** 2023-10-03

**Authors:** G. Ranzato, I. Lora, B. Aernouts, I. Adriaens, F. Gottardo, G. Cozzi

**Affiliations:** 1 University of Padova, Department of Animal Medicine, Production and Health (MAPS), Viale dell’Università 16, 35020 Legnaro, (PD) Italy; 2 KU Leuven, Department of Biosystems, Division of Animal and Human Health Engineering, Kleinhoefstraat 4, 2440 Geel, Belgium

**Keywords:** Behavior, Dairy cow, Heat stress, Precision livestock farming, Sensor data

## Abstract

Heat stress impairs the health and performance of dairy cows, yet only a few studies have investigated the diversity of cattle behavioral responses to heat waves. This research was conducted on an Italian Holstein dairy farm equipped with precision livestock farming sensors to assess potential different behavioral patterns of the animals. Three heat waves, defined as at least five consecutive days with mean daily temperature-humidity index higher than 72, were recorded in the farm area during the summer of 2021. Individual daily milk yield data of 102 cows were used to identify “heat-sensitive” animals, meaning the cows that, under a given heat wave, experienced a milk yield drop that was not linked with other health events (e.g., mastitis). Milk yield drops were detected as perturbations of the lactation curve estimated by iteratively using Wood’s equation. Individual daily minutes of lying, chewing, and activity were retrieved from ear-tag-based accelerometer sensors. Semi-parametric generalized estimating equations models were used to assess behavioral deviations of heat-sensitive cows from the herd means under heat stress conditions. Heat waves were associated with an overall increase in the herd’s chewing and activity times, along with an overall decrease of lying time. Heat-sensitive cows spent approximately 15 min/days more chewing and performing activities (*p* < 0.05). The findings of this research suggest that the information provided by high-frequency sensor data could assist farmers in identifying cows for which personalized interventions to alleviate heat stress are needed.

## Introduction

The effect of environmental heat on livestock species is a topic of growing concern, especially in light of the current climate change (Vitali et al. 2015). Future scenarios for the temperature-humidity index (THI) are not promising: Segnalini et al. (2013) forecasted an increase of THI in the Mediterranean area which will cause growing thermal discomfort to the animals, with negative consequences on their welfare, performance, health, and survival (Vitali et al. 2015). In addition to the overall increase in temperatures, heat waves (HWs) are becoming more and more frequent, intense, and extended (Beniston et al. 2007). In the next decades, the Mediterranean basin is expected to be dominated by increased droughts and HW (Gao and Giorgi 2008) to the point that it has been categorized as a global warming hotspot (Segnalini et al. 2013).

Lactating dairy cows produce a large quantity of metabolic heat, that under heat stress times is coupled with a compromised cooling capability because of environmental conditions (West 2003). This brings heat load in the cows to raise, causing an increase of body temperature and the inability to maintain thermal energy balance (West 2003; Becker et al. 2020). As adaptive response, the animals show physiological and behavioral changes (Islam et al. 2021): increased respiration rate (de Andrade et al. 2017; Becker et al. 2020), decreased lying time (Allen et al. 2015; Becker et al. 2020; Hut et al. 2022), increased shade utilization (Brown-Brandl et al. 2003; Becker et al. 2020), increased water intake (Coimbra et al. 2012; Becker et al. 2020), and reduced feed intake resulting in reduced milk yield (Bohmanova et al. 2007; Becker et al. 2020). Each of these adaptations aims at mitigating metabolic heat production and promoting the dissipation of body temperature (Islam et al. 2021). Furthermore, thermal stress may partially suppress the innate immune functions in lactating cows, leading to a higher risk of clinical diseases such as mastitis and metritis (Becker et al. 2020).

Monitoring dairy cows is crucial to identify and manage heat stress to limit its negative impact on welfare, health, and production (Hut et al. 2022). Nowadays, many sensor systems are commercially available to replace visual observation of the animals, which can be impractical in large commercial herds (Barriuso et al. 2018). Accelerometer-based systems are the most widely available and validated technology for continuous, real-time, and autonomous monitoring of core behaviors like eating, rumination, lying, and walking (Allen et al. 2015; Stygar et al. 2021; Islam et al. 2021). The information provided by sensor-based behavioral data can be used to assist farmers in the early identification of climate-related distress (Abeni and Galli 2017), minimizing the negative economic and welfare implications of heat stress.

Several studies have already investigated the main overall effects of heat stress on dairy cows’ behavior, as described above. However, to our knowledge, only Islam et al. (2021) analyzed changes in cows’ behavior based on the animals’ different responses to thermal stress. To further explore this topic, we retrospectively identified the “heat-sensitive” cows of a dairy herd based on their drop in milk yield associated with summer heat waves. Individual high-frequency sensor data were analyzed to detect different behavioral patterns of heat-sensitive animals and to explore the potential of sensor systems to early identify cows for which personalized interventions to alleviate heat stress are needed.

## Materials and methods

### Dataset

The study used data (Ranzato 2023) of 369 Holstein-Friesian cows (43% primiparous, 57% multiparous) from a dairy farm located in the Po Valley, Italy. The animals were housed in two barns equipped with high-volume low-speed horizontal ceiling fans and with cooling showers along the feeding alleys (“new ventilation system”) in the period between 25 days in milk (DIM) and 280 DIM. During the post-partum days and in the late lactation phases, the cows were moved to two barns equipped with only old vertical fans (“old ventilation system”). For the period from 28 March to 30 September 2021 covered by the study, the animals were fed with total mixed rations based on maize silage, grass silage, maize and soybean meals.

Behavioral data regarding lying (LIE), chewing (CHEW), and activity (ACT) times were collected by an ear-tag-based accelerometer (Smartbow GmbH, Weibern, Austria) that registered the time budgets of each cow by measuring head and ear movements (Krieger et al. 2019). The ear tag captured and sent acceleration data once per second (1 Hz); daily minutes of LIE (lying + standing = 1440 min/day), CHEW (chewing + “not chewing” = 1440 min/day), and ACT (activity + inactivity = 1440 min/day) were used in the study. Individual cow health events and individual daily milk yield (MY) data, automatically recorded in the milking parlor, were also retrieved from the farm databases.

Climate data were restored from the online archive of the local environmental protection agency (Agenzia Regionale Protezione Ambiente 2022), by referring to the nearest weather station to the farm (7 km of distance). The average daily temperature and average daily relative humidity were used to compute average daily THI according to the equation by Kelly and Bond (1971). A HW is generally described as a prolonged period of excessively hot weather, but no official definition is available (Maggiolino et al. 2022). Commonly, a THI of 72 is considered the threshold after which milk production starts to decrease in Holstein cows because of thermal discomfort (Segnalini et al. 2013; Heinicke et al. 2018). In this study, a HW was then defined as a period of at least 5 consecutive days (Frich et al. 2002) with a mean daily THI ≥ 72. If successive HW were less than 3 days apart from each other, they were considered as one HW (therefore, one HW could contain days with THI < 72). Three HW were identified in the period of summer 2021 (June–September): from 11/06 (dd/mm) to 18/06; from 17/07 to 31/07; and from 08/08 to 16/08. Table 1 gives an overview of the meteorological characteristics of each HW.

**Table 1:** Characteristics of the three heat waves (HWs) recorded during the summer of 2021: mean daily temperature (T), mean daily relative humidity (H), mean daily temperature-humidity index (THI), minimum daily THI, maximum daily THI, number of cows (overall *n* = 102), percentage of heat-sensitive cows (i.e., cows that started one or more perturbations of the lactation curve during the heat wave)

	T (°C)	H (%)	THI			Cows (n)	Heat-sensitive cows (%)
			mean	min.	max.		
HW 1 (11/06^a^–18/06)	25.6	61.6	73.8	72.1	75.6	85	12
HW 2 (17/07–31/07)	24.8	75.3	74.1	70.9	75.8	66	18
HW 3 (08/08–16/08)	25.5	72.2	74.8	72.0	78.2	57	37

^a^dd/mm

Data mining, processing steps, and statistical analyses were carried out with RStudio software (R version 4.1.2; RStudio PBC, Boston (MA), USA).

### Heat-sensitive cows

**Fig. 1:**
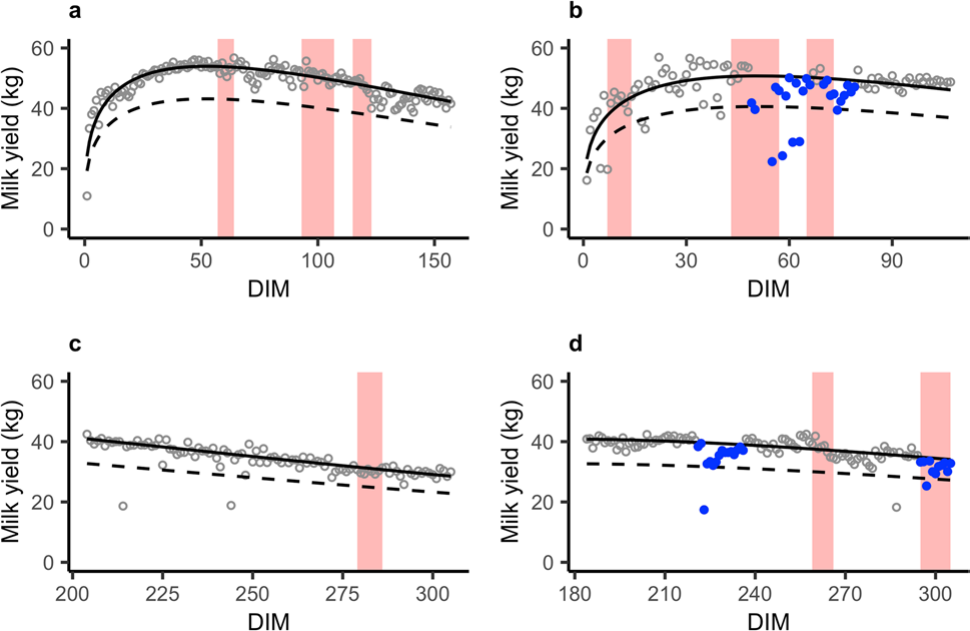
Estimated theoretical milk production curve (solid line; respective 80% in dashed line) and identified perturbations (blue dots) for different lactations during the observation period (**a** 1 ≤ DIM ≤ 157; **b** 5 ≤ DIM ≤ 180; **c** 204 ≤ DIM ≤ 305; **d** 221 ≤ DIM ≤ 305). The pink-colored areas identify the heat waves during the observation period

Daily MY data were used to identify the cows that were more sensitive to thermal heat. Given that at any production level dairy cattle show an inverse relationship between milk yield and heat stress (Ravagnolo et al. 2000; West 2003; Becker et al. 2020), we assumed “heat-sensitive” cows to be the ones that started at least one consistent drop in milk production during a HW. Following the work by Adriaens et al. (2021), drops in milk production were identified as perturbations in the lactation curve compared with the theoretical production for that lactation (i.e., potential milk production when no disturbances are present). Unlike in Adriaens et al. (2021), we did not have access to complete lactations data due to the restricted time period of the study. Therefore, lactations were selected based on the following criteria: (i) MY data were available from before DIM 30 for at least 100 days or (ii) MY data were available from beyond DIM 150 for at least 50 days. These filters were necessary to grasp a proper image of the lactation curves in the observation period. We removed records beyond DIM 305 for standardization purposes, because the last part of the lactation curve can be influenced by the gestation stage and feed changes towards dry-off (Adriaens et al. 2021; Ben Abdelkrim et al. 2021). After data editing to remove recording errors (e.g., MY = 0 kg/d), we kept only lactations with no more than 2 gaps of at most 5 days each. To determine the theoretical shape of the lactation curves of the 108 cows left in the dataset, a Wood model (Wood 1967) was iteratively fitted on the MY data of each animal (for more details, see Adriaens et al. 2021). Next, the periods classified as perturbations of the milk production curve were identified as at least 5 days of successively negative residuals with at least 1 day of MY lower than 80% of the theoretical curve. To illustrate this methodology, MY data of 4 cows are plotted in Fig. 1, and MY perturbations, if present, are highlighted in blue.

Cows with registered pathologies or health events that influenced their lactation curve (e.g., mastitis) were removed from the dataset to retain only animals with MY perturbations potentially due to heat stress. Nonetheless, for some lactations, perturbations of MY were detected also outside HW. These were handled with the following criteria: (i) when they started before a HW and overlapped with the HW itself, the respective records during HW days were removed to consider only perturbations originated under thermal stress; (ii) when they started during a HW and continued under the next HW, they were kept as prolonged heat stress effects; and (iii) when they were completely outside HW, they were kept as perturbations caused by unknown reasons. Two examples of cows found to be heat-sensitive to one or more heat waves are shown in Fig. 1b and d.

### Data processing and statistical analysis

A categorical variable identifying the stage of the lactation was created according to the observed time period of each animal: “early lactation” when DIM ≤ 100, “mid lactation” when 100 < DIM ≤ 200, and “late lactation” when DIM > 200 (Niozas et al. 2019). A binary variable was created to indicate the type of barn where the cows were housed (i.e., “old ventilation system” vs. “new ventilation system”). On specific dates, the sum of daily minutes of each behavior (i.e., LIE, CHEW, ACT) with its complementary (i.e., standing, not chewing, inactivity) was lower than 1440 min/day, probably due to recording errors: when the sum was below 1320 min/day (i.e., more than 2 h of the day were missing), that record was deleted from the dataset; when the sum was between 1320 and 1439 min/day, the respective values were reproportioned to sum to 1440 min/day. The final dataset contained 102 cows with a number of observations ranging between 48 and 159 days of data.

To detect different behavioral trends of heat-sensitive cows, we first used linear mixed-effects models, which are traditionally applied for analyzing longitudinal data. They produced non-normal residuals, even when using transformations of the response variables (i.e., LIE, CHEW, ACT). Accordingly, we used a semi-parametric technique that handles repeated measures, referred to as generalized estimating equations (GEE; R package “geepack,” Halekoh et al. 2006). Generalized estimating equations are used to estimate the parameters of a (generalized) linear model specifying a working correlation structure that accounts for within-subject correlation of the response variable (Hardin and Hilbe 2013). Different correlation structures can be specified, including independence of observations, exchangeable correlation, first-order autoregressive structure, and unstructured correlation; the most appropriate working correlation structure is the one that produces the smallest correlation information criterion (CIC; Hin and Wang 2009). Generalized estimating equations models are population average models, meaning that the estimated effects are interpreted as for (generalized) linear models but at “population” level (Hubbard et al. 2010).

The GEE models for the different behaviors were specified as follows:

1 $${\mu}_{\mathrm{i}\mathrm{j}}={\beta}_0+{\beta}_1{multiparous}_{\mathrm{i}}+{\beta}_2 mid\ {lactation}_{\mathrm{i}\mathrm{j}}+{\beta}_3{late\ lactation}_{\mathrm{i}\mathrm{j}}+{\beta}_4 new\ {ventilation\ system}_{\mathrm{i}\mathrm{j}}+{\beta}_5{HW}_{\mathrm{i}\mathrm{j}}+{\beta}_6{perturbation}_{\mathrm{i}\mathrm{j}}+{\beta}_7{HW}_{ij}\ast {perturbation}_{\mathrm{i}\mathrm{j}},$$

where *μ*_ij_ represents the mean of the response variable *y*_ij_ (*j* = 1, …, 159 daily measurement of cow *i* = 1, …, 102) corresponding to LIE, CHEW, or ACT times. The variance of *y*_ij_ is a function of a known variance function *v* of the mean and a known scale parameter (*V*(*y*_*ij*_) = *v*(*μ*_*ij*_)*ϕ*), accounting for within-subject correlation of the observations. The information on the HW was expressed by a binary time-dependent variable (“no HW” as reference class, and “HW”), as well as for the presence of drops in the milk curve (“no perturbation” as reference class, and “perturbation”). The interaction term referred to heat-sensitive cows, i.e., cows experiencing one or more MY perturbations (*perturbation* = 1) started during a HW (*HW* = 1). The reference categories of the variables used to adjust the comparison between cows were “primiparous” for the parity information, “early lactation” for the variable indicating the stage of the lactation, and “old ventilation system” for the variable distinguishing the barn based on the type of ventilation system.

The effect of each variable in Eq. 1 was quantified by the estimation of the related regression parameter. The effect of *HW* and *perturbation* had to be averaged over the levels of the other variable involved in the interaction term (e.g., *HW* effect $={\hat{\mu}}_{HW}-{\hat{\mu}}_{no\ HW}={\hat{\beta}}_5+{\hat{\beta}}_7 perturbation=\left\{{\hat{\beta}}_5+\left({\hat{\beta}}_5+{\hat{\beta}}_7\right)\right\}/2$). The behavioral variation of heat-sensitive cows with respect to the farm means during HW (i.e., *HW* = 1, *perturbation* = 1 vs. *HW* = 1, *perturbation* = 0) was estimated by ${\hat{\beta}}_6+{\hat{\beta}}_7$.

## Results and discussion

Three HW were identified during the summer of 2021 (June–September) in the area of the Po Valley where the farm was located. The percentage of heat-sensitive cows in the herd, i.e., the percentage of cows that experienced one or more MY perturbations during a HW, increased from the first to the last HW (Table 1). This result was somehow expected, as the last HW (from 8 to 16 August) was the most severe with a maximum daily THI of 78.2. A THI ≥ 75, in fact, generates alarming conditions for both the welfare and performance of dairy cows (Segnalini et al. 2013). However, we cannot exclude that a heat stress carry-over effect may also have played a role in the increase of the percentage of heat-sensitive cows as a function of the number of HW (Herbut et al. 2018).

Sensor technologies provide opportunities to constantly monitor dairy cattle behavior, and they may assist farmers in the early identification of thermal stress symptoms (Abeni and Galli 2017; Hut et al. 2022). To our knowledge, the ear-tag-based accelerometer sensor used in this study has been validated for chewing monitoring (Borchers et al. 2016; Reiter et al. 2018), but no references could be found for lying and activity times in dairy cows. Only Roland et al. (2018) reported that the ear tag reached a satisfying accuracy in detecting posture (i.e., lying vs. standing) and a substantial agreement for some activities in dairy calves. The average sensor-based LIE time of the herd, recorded in the period 28 March–30 September 2021, was 693 min/day, while mean CHEW and ACT times were respectively 596 min/day and 1097 min/day (Table 2). Data available in the literature show that lactating dairy cows spend 660 to 840 min/day lying down under thermoneutral conditions (Becker et al. 2020). Chewing time may vary across dairy herds as chewing activity can be affected by physical properties of the diet (Beauchemin et al. 2003) and selective feed intakes of the animals (Maulfair et al. 2010), but also by farmers’ decisions on cows’ grouping (Grant and Albright 2001). Even activity is a rather variable measure for which it is difficult to define a reference range, as it can vary due to barn design, herd management, and especially type of sensor system used for its recording.

**Table 2:** Minimum, first quartile (Q_1_), median, mean, third quartile (Q_3_), and maximum of the behavioral data retrieved from ear-tag-based accelerometer sensors (lying, LIE; chewing, CHEW; activity, ACT)

	LIE (min/d)	CHEW (min/d)	ACT (min/d)
min.	156	125	442
Q_1_	592	537	1023
median	700	592	1089
mean	693	596	1087
Q_3_	801	650	1153
max.	1212	1085	1426

Results from the three GEE models fitted on LIE, CHEW, and ACT times are reported in Table 3. No model simplification strategy was applied. The working correlation structure that produced the smallest CIC was, for all the models, the exchangeable structure, meaning that all pairs of observations within a subject could be considered equally correlated.

**Table 3:** Results from the generalized estimating equations models on cows’ daily minutes of lying (LIE), chewing (CHEW), and activity (ACT): estimated parameters related to the different variables (coeff.), standard errors (SE) in brackets, and corresponding observed levels of significance (*p*)

	LIE (min/d)		CHEW (min/d)		ACT (min/d)	
	coeff. (SE)	*p*	coeff. (SE)	*p*	coeff. (SE)	*p*
*β*_0_ intercept	679 (18.7)	***	557 (9.19)	***	1096 (12.7)	***
*β*_1_ multiparous	86.7 (22.1)	***	40.0 (13.1)	**	−54.8 (13.5)	***
*β*_2_ mid lactation	−3.70 (9.78)	n.s.	5.95 (5.89)	n.s.	15.2 (6.45)	*
*β*_3_ late lactation	−18.7 (11.2)	n.s.	4.75 (7.30)	n.s.	6.15 (8.45)	n.s.
*β*_4_ new ventilation system	−14.4 (8.36)	n.s.	7.32 (5.84)	n.s.	1.05 (8.12)	n.s.
*β*_5_ HW^a^	−37.0 (3.30)	***	38.8 (3.92)	***	43.8 (3.11)	***
*β*_6_ perturbation	12.0 (7.53)	n.s.	−10.3 (4.58)	*	−23.9 (6.30)	***
*β*_7_ HW ∗ perturbation	−9.87 (12.5)	n.s.	25.5 (13.1)	*	39.4 (21.3)	**

*** *p* < 0.001, ** *p* < 0.01, * *p* < 0.05, n.s. *p* ≥ 0.05

^a^Heat wave

Cow’s parity affected all the recorded behaviors (Table 3). Multiparous cows had longer LIE and CHEW times being less active than primiparous ones. Behavioral differences across parities have been described earlier, although mostly focused on the animals’ transition period (Azizi et al. 2010; Neave et al. 2017). As an effect of hierarchical differences between primiparous and multiparous cows, younger animals entering the milking herd for the first time spend less time lying down and increase their daily activity.

Overall, the average LIE time of the herd decreased during HW periods by 42 min/day. By standing, in fact, cattle expose a greater body surface to the air which helps heat loss due to the convection phenomenon (Allen et al. 2015). Chewing time increased by 52 min/day under prolonged periods of environmental heat. This result may seem in contradiction with some works affirming a tendency of chewing activity to decrease under heat stress conditions (Karimi et al. 2015; Maia et al. 2020). Considering chewing time as the summation of rumination and eating times (Perdomo et al. 2020), the increase of CHEW found in this study could be determined by an increase of eating time due to the presence of cooling systems along the feeding alleys. However, the accuracy of accelerometer ear tags in monitoring chewing activity can be variable depending on the conditions of use, and it can be biased by other movements of the animal’s head (Beauchemin 2018). The average ACT time increased by 64 min/day during HW. The ear tag recorded a cow being active when the animal was actively moving either standing or lying down. Therefore, ACT could include walking, exploring, drinking, urination, defecation, grooming, head swing, and estrus expression (Zambelis et al. 2019; Becker et al. 2020). Consistent with this finding, Abeni and Galli (2017) reported higher daily activity times associated with higher THI exposure in dairy cows: the animals tend to have more frequent movements of the head, recorded by the sensor, when the environmental temperature increases (Cook et al. 2007). Brzozowska et al. (2014) stated that also the number of steps per day increases during heat stress periods.

Focusing on the heat-sensitive cows (${\hat{\beta}}_7$, *HW* ∗ *perturbation* effect in Table 3), their behavioral patterns were similar to those of all the cows exposed to HW, but more severe variations were detected for CHEW and ACT times. Heat-sensitive cows chewed 15 min/day more and were 16 min/day more active with respect to the herd means during HW. To our knowledge, only Islam et al. (2021) compared the behavior of “heat-susceptible” and “heat-tolerant” dairy cows by making a distinction based on their panting scores. These authors compared sensor-based eating, rumination, and lying times of the two groups of cows during 3 different HW events, but without involving thermoneutral conditions in the study. They found that heat-susceptible cows spent more time eating (*p* < 0.001) and less time lying down (*p* = 0.04) compared to heat-tolerant cows, suggesting that heat-tolerance can be at the expense of reduced production either by inborn genetic merit or by adaptive reduced feed intake. Similarly, we could assume that heat-sensitive cows increased their daily eating time resulting in longer chewing times.

**Fig. 2:**
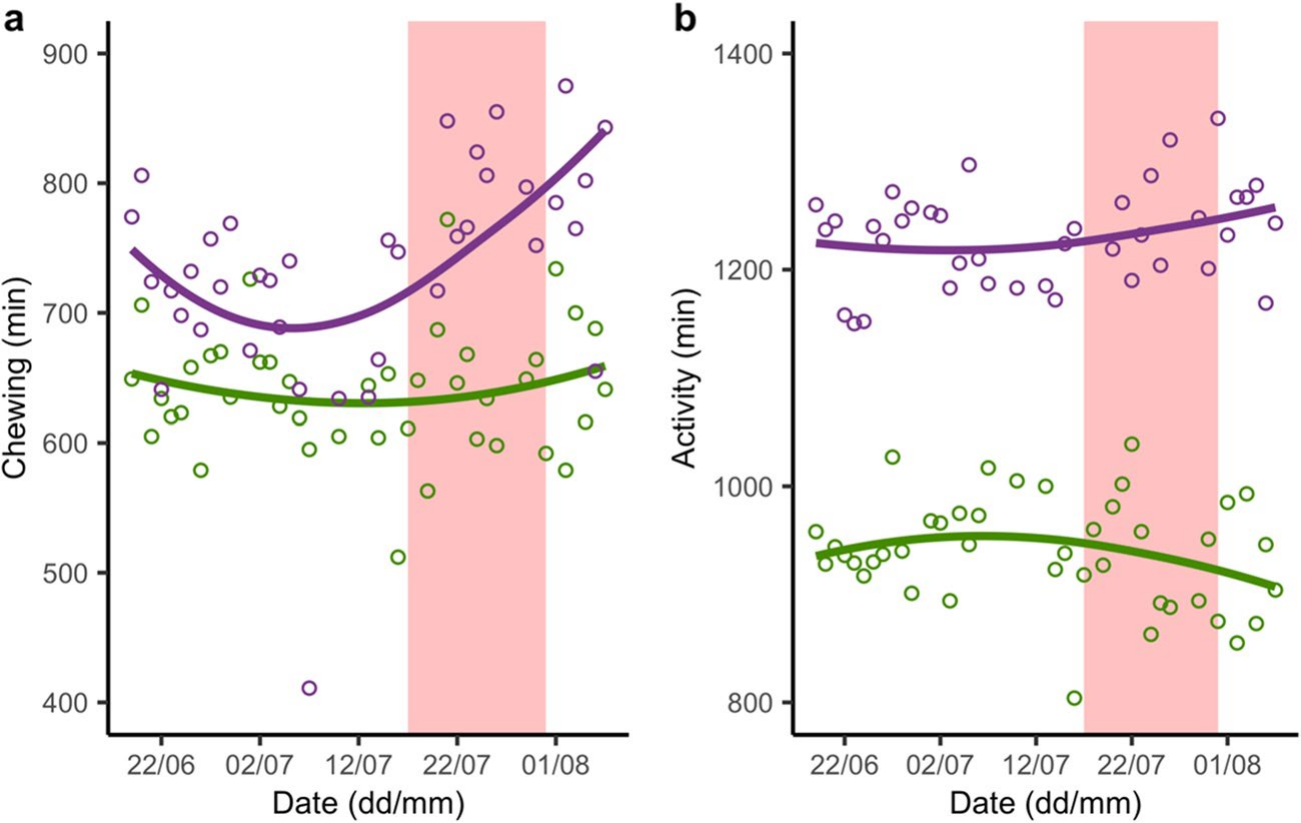
Chewing (**a**) and activity (**b**) patterns of two cows during one period of summer 2021. One cow (green line) was not sensitive to the heat wave from 17 to 31 July (pink-colored area), the other (purple line) was sensitive to the same heat wave

Our results indicate that cows belonging to the same herd, therefore under the same environmental and management conditions, can have different behavioral adaptations to heat stress. Fig. 2 is an example of different CHEW (Fig. 2a) and ACT (Fig. 2b) patterns of two cows of the herd, one of which resulted to be sensitive to the HW from 17 to 31 July. The identification of the more sensitive animals to thermal distress through the monitoring of their behavioral responses could allow targeted interventions by the farmers to alleviate heat stress symptoms. Farmers could decide to create specific groups of heat-sensitive cows to be housed in areas where the cooling is more effective, or to adjust their feeding schedule as limiting feed availability during the hottest hours of the day can reduce heat stress (Davis et al. 2003). In parallel, they could select “heat-tolerant” cows (i.e., the ones that did not experience any MY perturbations during HW) for breeding purposes and optimize breeding schemes and culling decisions (Ranzato et al. 2022). Recent studies have in fact shown that selection for heat-tolerant cows’ genotypes is feasible and leads to improvements in milk production and feed intake during and after heat stress events (Liu et al. 2017).

As sensors systems are in continuous progress in the dairy farming sector, it is possible that heat stress sensitivity will be even more accurately assessed combining behavioral information with additional parameters automatically detected. Chen et al. (2018) found that cows more sensitive to thermal stress have higher rectal temperature than less sensitive ones, along with upregulated metabolisms and downregulated neurodegenerative disease pathways. Liu et al. (2017) obtained differences in lipid concentration of milk between heat-sensitive and heat-tolerant cows during heat stress. Finally, Herbut et al. (2018) affirmed that the time of the year and the breed of the cows may have a big impact on when the animals become sensitive to increasing heat loads.

Under heat stress conditions, a consistent decrease in daily milk production is usually registered 48 h after the thermal stress onset (Spiers et al. 2004). The advantage of referring to high-frequency behavioral data for heat stress detection is that the sensor system could immediately give an alarm when, for example, chewing and activity times overpass specific thresholds, thus preceding the actual milk yield drop. Further research involving more farms and covering more years of recorded behavioral data could be useful to lay the foundations for a decision-support tool for dairy farmers.

## Conclusions

This study aimed at exploring differences in Holstein-Friesian dairy cows’ lying, chewing, and activity times, recorded by ear-tag-based accelerometer sensors, when exposed to summer heat waves. “Heat-sensitive” cows were identified by the presence of one or more drops in milk yield that started during a given heat wave. The percentage of cows that resulted to be sensitive to heat stress increased progressively from the first to the last heat wave detected during the observation period. Heat-sensitive cows revealed significant deviations from the herd mean behaviors during heat waves. In particular, they spent more daily time chewing and being active, probably in the attempt to better cope with the environmental heat. The identification of more sensitive animals to thermal distress through the automatic monitoring of their behavior could allow targeted interventions by the farmers to alleviate heat stress symptoms.

## Data Availability

Data openly available in a public repository that issues datasets with DOIs.
